# Effect of SARA Fractions on Fatigue Properties of Hard Asphalt

**DOI:** 10.3390/ma17112713

**Published:** 2024-06-03

**Authors:** Jinyi Jiang, Fei Lu, Xiaogang Guo, Peiwen Hao, Wei Wang, Jincheng Yu

**Affiliations:** 1 Key Laboratory of Road Structure & Material, Ministry of Communication, Chang’an University, Xi’an 710064, China; chiangchingyii@outlook.com; 2 Zhejiang Engineering Research Center of Digital Highway Applied Technology, Zhejiang Institute of Communications, Hangzhou 311112, China; 3 China Communications Construction Company Infrastructure Maintenance Group Co., Ltd., Beijing 100011, China; 4 School of Concrete and Construction Management, Middle Tennessee State University, Murfreesboro, TN 37132, USA

**Keywords:** hard asphalt, SARA fractions, rutting factor, fatigue performance, strain sensitivity

## Abstract

The fatigue performance of hard asphalt is an important factor that affects the service life of asphalt pavement. In order to comprehensively explore the influence of chemical components on the fatigue performance of hard asphalt, and to eliminate the chemical instability between the microstructure of asphalt from different oil sources, seven kinds of hard asphalt were designed and prepared with saturates, aromatics, resins, and asphaltenes (SARA) extracted from the same hard asphalt. Rheological, time sweep and linear amplitude sweep (LAS) tests were carried out to evaluate the fatigue properties. The results show that the complex modulus of asphalt binds increased rapidly with an increase of asphaltene and resins and that the colloidal structure was strengthened, which would increase the fatigue factor. In the time sweep test, the strength of the colloidal structure significantly affected the fatigue life, and the fatigue life was different under different test stresses. In the viscoelastic continuum damage (VECD) model, the cumulative damage was related to the modulus, while with the increase of asphaltene and resins, the fatigue life showed a trend of first increasing and then decreasing. The linear regression analysis showed that the fatigue life of hard asphalt had a good correlation with strain sensitivity. This study investigated the applicability of different fatigue evaluation methods and revealed the influence of four components on the fatigue properties of hard asphalt. The results provide significant insights in the improvement of the fatigue performance of both hard asphalt and corresponding mixtures.

## 1. Introduction

Due to the repeated actions of vehicle loads, the fatigue damage generated inside the pavement material structure has become one of the main types of pavement distress, and the fatigue failure caused by fatigue damage has become a major failure mode restricting the service life of asphalt pavement [1,2]. The fatigue performance of asphalt mixture directly affects the service life of asphalt pavement. Previous studies have shown that the fatigue failure of asphalt binder acts as a crucial role in determining the fatigue life of the mixture [3], and fatigue cracks generally initiate and propagate within the binder or mastic phase of asphalt mixtures. Because of the significant contributions of asphalt binders to the fatigue performance of asphalt pavement [4,5], an assessment of the fatigue performance of the asphalt binder is pivotal [6].

Selecting an appropriate asphalt binder is an important method by which to prevent premature fatigue failure of asphalt pavement. In the field of engineering, Enrobé a Module Élevé (EME), is known, in the French standard, as the high modulus asphalt mixture in the base and sub-base layer of the pavement structure. It has a larger amount of asphalt, thicker asphalt film and smaller porosity than conventional asphalt mixtures; can achieve excellent durability and fatigue resistance; and has been used on a large scale [7,8]. The asphalt binder used in EME, which needs a penetration grade at 25 °C lower than 25 (0.1 mm), is also known as hard asphalt [9,10,11].

The research on improved asphalt fatigue tests is an ongoing effort and has been introduced over the past decades within the framework of current performance grade (PG) asphalt specifications. A dynamic shear rheometer (DSR) is employed for evaluating the viscoelastic properties of an asphalt binder in terms of fatigue potential, and the linear viscoelastic parameter G*sinδ, known as the fatigue factor, is used to quantify asphalt fatigue resistance [12]. To establish a better rating of the role of binders in mixture fatigue, the parameter N*_f_*, defined as the number of cycles required to propagate cracks, is recommended to replace the binder parameter G*sinδ through the time sweep (TS) test, which was proposed by Bahia during NCHRP 9-10 [13,14]. Under the TS test mode, the direct application of cyclic loading at sufficient stiffness levels, along with fixed frequency and amplitude in either controlled stress or controlled strain mode, was approved and the relevant fatigue performance indicators measured. A significant amount of research has been devoted to defining fatigue failure. The simplest fatigue failure definition used in asphalt material tests is 50% loss in stiffness or pseudo stiffness [15,16,17,18,19,20,21,22]. The Glover–Rowe parameter is a function of two rheological properties, and complex shear modulus (G*) and phase angle (delta) are measured at 15 °C and a frequency of 0.005 radians/second in the DSR, which needs more test time than the TS test [23].

As the TS test requires a long testing time, an accelerated testing process named the linear amplitude sweep (LAS) test has been used in asphalt material testing, based on the simplified viscoelastic continuum damage (VECD) theory [24,25,26]. The LAS test is an accelerated amplitude sweep procedure that is used instead of a fatigue-type test in either controlled stress or controlled strain mode [27]. The test data interpretation is then used to predict the fatigue life within a significantly shorter time compared with the TS test. Thus, the method has been widely accepted and has been found to correlate well with the fatigue behavior of asphalt pavement [28].

In the research on the fatigue performance of hard asphalt, a considerable number of findings have yielded meaningful research results. Zou’s research has pointed out that different oil sources and different production processes of hard asphalt have different effects on the fatigue life of asphalt mixture [29]. Quan revealed that epoxidized rapeseed oil has a positive impact on the fatigue performance of modified hard asphalt binders [30]. Qin has indicated that the fatigue performance of hard asphalt is much lower than penetration grade 70 asphalt in the DSR fatigue test under a strain control model [31]. It was found that the strain level in a hard asphalt mixture would be reduced due to the larger stiffness modulus, and that it possessed better fatigue resistance than conventional mix [32,33,34].

It should be noted that the macroscopic properties of asphalt are closely related to the chemical components of asphalt. Some studies on the influence of the chemical components on the fatigue properties of hard asphalt have been carried out from the perspective of chemical composition [35,36]. In order to better understand the influence of chemical components on the fatigue properties of hard asphalt, this study employed saturates, aromatics, resins, and asphaltenes (SARA), separated from the same hard asphalt by solvent elution method, to customize and design seven kinds of hard asphalt samples according to the related literature [37,38]. Additionally, rheological test, time sweep test and LAS test were conducted by DSR to investigate the fatigue characteristics through a comparative analysis of chemical components and fatigue test results. The research results of this paper suggest that the relative proportions of the four components should be considered when producing hard asphalt, such as when utilizing the blending method, so as to improve the strain sensitivity of hard asphalt, which has important engineering value when improving the fatigue properties of asphalt and EME mixtures.

## 2. Materials and Experiments

### 2.1. Materials

**Figure 1:**
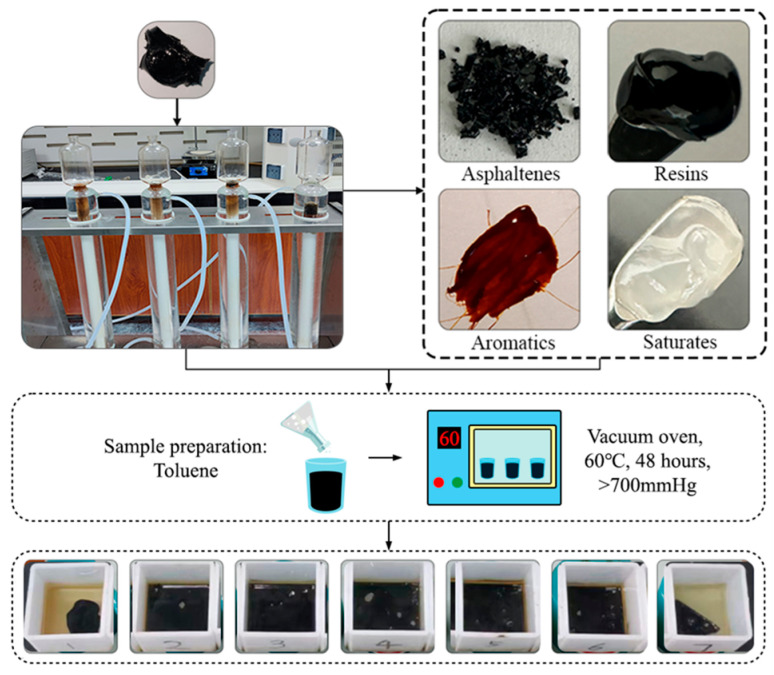
The preparation process of seven blend binders.

**Figure 2:**
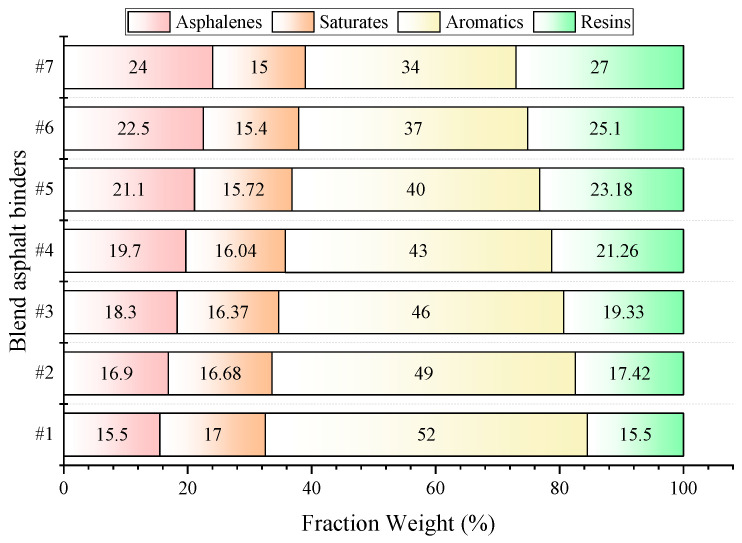
SARA Fraction contents of seven blend asphalt binders.

In this work, the compatibility stability between different asphaltenes and resins should be eliminated [39]. According to the asphalt chemical component test method (T0618) in Standard Test Methods of Bitumen and Bituminous Mixtures for Highway Engineering (Issued by Ministry of Transport, Beijing, China) [40], SARA fractions were separated from the same virgin hard asphalt, as shown in Figure 1. Seven kinds of asphalt binders were designed using the SARA fractions referring to the relevant literature, which were then blended with toluene solution in a ceramic mold and dried at 60 °C for 48 h in a vacuum oven for subsequent tests, as shown in Figure 2. Table 1 shows the properties of virgin hard asphalt and the results of SARA fractions.

### 2.2. Experiments

#### 2.2.1. Dynamic Shear Rheological (DSR) Tests

The rheological properties were determined by DSR (Marlvern Kinexus, Malvern Instrument Ltd., Malvern, UK) in order to conduct the complex modulus G* and the phase angle δ, and the temperature ranged from 40 to 82 °C in accordance with the ASTM D6373 [41], at intervals of 6 °C at 10 rad/s. The strain level was 12%, the gap was set to 1 mm and the diameter of the plates was 25 mm. The rutting factor G*/sinδ and fatigue factor G*sinδ could then be calculated [42].

#### 2.2.2. Time Sweep (TS) Test

The time sweep test under constant stress was used to study the fatigue damage characteristics of asphalt under cyclic loading. When the load amplitude was too small, the test would take too long and the specimen might not even suffer any fatigue damage. When the load amplitude was too large, the fatigue damage would be inflicted too quickly, making the fatigue life too small and the dispersion of the test results too large. Therefore, the appropriate load amplitude should be selected for the time sweep test. Considering the test duration and data stability, and referring to the research results [43], the stress control mode was selected. The stress amplitude was 0.1 Mpa and 0.2 Mpa, the parallel plate was 8 mm, the plate spacing was 2 mm, the oscillation frequency was 10 Hz, and the test temperature was 30 °C.

#### 2.2.3. Linear Amplitude Sweep (LAS) Test

The linear amplitude sweep (LAS) test used a DSR to assess the fatigue behavior of asphalt binders by the application of systematically increasing load amplitudes (0–30%) over the course of 3100 cycles at constant test temperature and loading frequency following the AASHTO TP 101-14 [26]. In this work, the LAS test was conducted under oscillatory shear in the strain-controlled mode at a frequency of 10 Hz at temperatures of 25 °C, 30 °C, and 35 °C and the gap and diameter of the plates were set to 2 mm and 8 mm, respectively. The LAS test consisted of two steps. Firstly, the frequency sweep test was conducted with the strain value (0.1%) in a range of frequencies from 0.2 to 30 Hz to determine the rheological properties of undamaged binder. Secondly, 3100 oscillatory shear load cycles under linearly increasing amplitudes were conducted on the binder to accelerate the fatigue damage. After that, the continuum damage approach of the viscoelastic continuum damage (VECD) [44] model was used to calculate the fatigue resistance in accordance with rheological properties and amplitude sweep test by following Equations (1)–(7).

The damage accumulation was calculated by Equation (1)

(1) $$D\left(t\right)≅{\sum_{i=1}^{N}\left[\pi I_{D}\gamma_{0}^{2}\left(\left|G*\right|\sin\delta_{i-1}-\left|G*\right|\sin\delta_{i}\right)\right]}^{\frac{\alpha }{1+\alpha }}{\left(t_{i}-t_{i-1}\right)}^{\frac{1}{1+\alpha }}$$

where *D*(*t*) represents the cumulative damage of asphalt; $I_{D}$ represents the initial value of $\left|G*\right|$ obtained from the 1.0% applied strain interval, MPa; $\gamma_{0}$ is equal to the strain exerted by the test procedure for the data point at a certain time, %; $\left|G*\right|$ refers to the complex shear modulus, MPa; α corresponds to the parameter of rheological property, dimensionless; and t represents the testing time, s.

The binder fatigue performance parameter *N_f_* can be calculated as follows in Equation (2):

(2) $$N_{f}=A_{35}{\left(\gamma_{max}\right)}^{-B}$$

where $\gamma_{max}$ is the maximum expected binder strain for a given pavement structure, usually a value of 2.5% or 5%; *A* and *B* are parameters for the binder fatigue performance model shown as Equations (3) and (4), respectively; *f* is the loading frequency (10 Hz); *k* is the coefficient shown as Equation (5); *D_f_* is defined as that which corresponds to a 35% reduction in undamaged |G*|·sinδ· (*C*_0_), the calculation of which is obtained by Equation (6). The relationship between |G*|·sinδ and *D*(*t*) can be fit to the power law relationship shown as Equation (7), *C*_0_ is the average value of |G*|·sinδ from the 0.1% strain interval, and *C*_1_ and *C*_2_ are curve fit coefficients.

(3) $$A_{35}=\frac{f{\left(D_{f}\right)}^{k}}{k{\left(\pi I_{D}C_{1}C_{2}\right)}^{\alpha }}$$

(4) B=2α

*k* = 1 + (1 − *C*_2_)*α*. (5)

(6) $$D_{f}=\left(0.35\right){\left(\frac{C_{0}}{C_{1}}\right)}^{\left(\frac{1}{C_{2}}\right)}$$

(7) $$\left|G*\right|\cdot \sin\delta =C_{0}-C_{1}{\left(D\right)}^{C_{2}}$$

## 3. Results and Discussion

### 3.1. Rheological Properties Test Results

**Figure 3:**
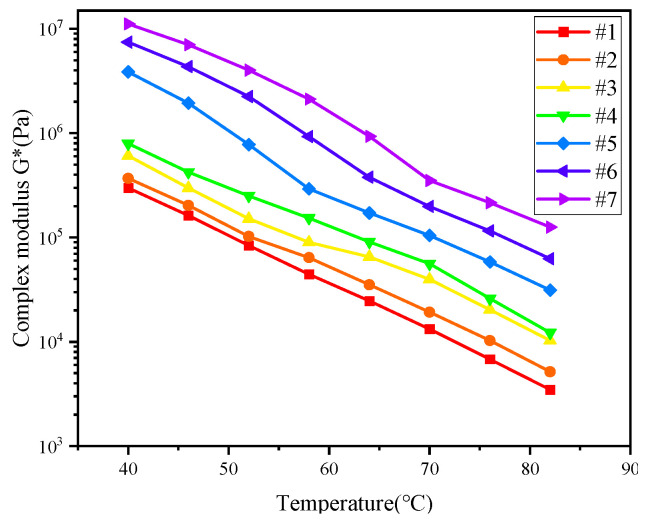
Complex shear modulus.

**Figure 4:**
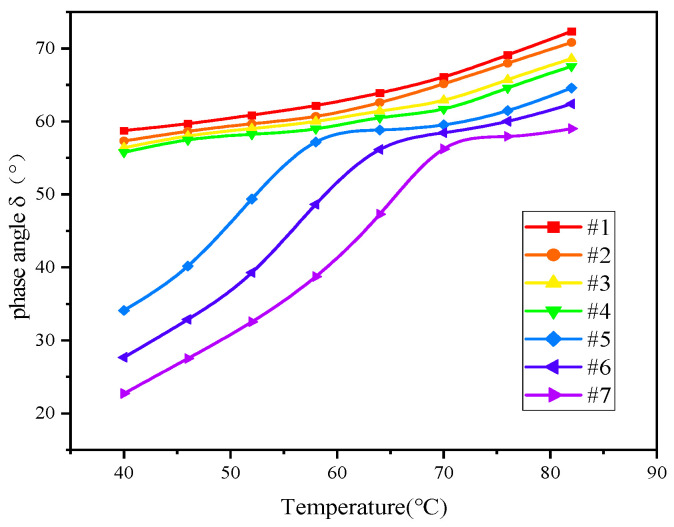
Phase angle.

The temperature sweeping tests were performed with a constant frequency of 10 rad/s [45], and the results of the temperature sweep tests of the seven asphalt binders are shown in Figure 3 and Figure 4. The phase angle reflects the ratio of the flexibility and viscous components of the asphalt binder. For viscoelastic materials, the phase angle is distributed between 0° and 90°, with 0° being a purely elastic material, and 90° purely viscous. The greater is δ, the greater part of a deformation that cannot be recovered and the more permanent a deformation appears to be. As shown in Figure 3 and Figure 4, with increasing temperature, the complex shear modulus decreased and the phase angle increased, indicating that the binder gradually transformed from a viscoelastic fluid to a viscous fluid. At the same temperature, the modulus increased with increasing sample number in the order of #7 > #6 > #5 > #4 > #3 > #2 > #1, which means that, with increasing resin and asphaltene content, the complex modulus of the binder increased significantly. An increase in resins and asphaltenes could effectively enhance the stiffness and improve the rutting resistance due to the presence of strong intermolecular interactions between molecules consisting of heteroatoms [46]. The spatial network structure built by resins and asphaltenes could directly enhance the strength of the asphalt colloid structure. Moreover, for samples #5 to #7, as the test temperature increased, the complex modulus decreased dramatically, and the phase angle increased sharply. These results imply that the increase in resins and asphaltenes might, to a certain extent, cause the instability of the colloid structure, resulting in an increase in the temperature sensitivity [47,48].

**Figure 5:**
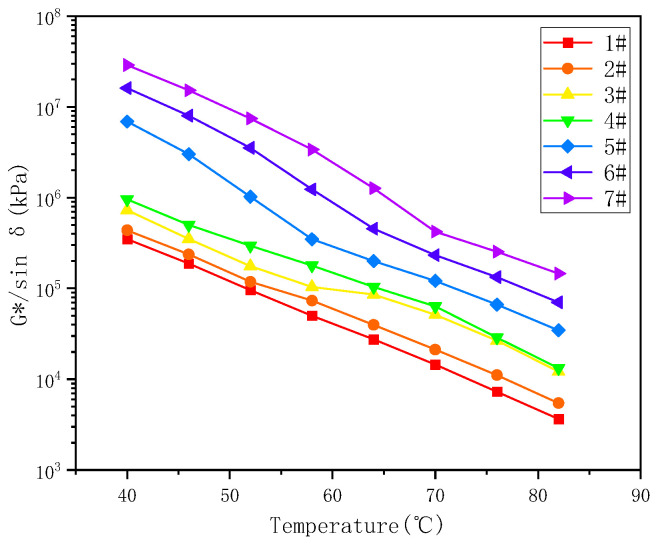
Variation of G*/sinδ versus temperature.

The anti-rutting performance of the asphalt binder can be evaluated according to rutting factor (G*/sinδ). At high temperatures, higher G*/sinδ values are preferred to reduce the energy dissipation due to repeated loading. The less energy dissipated per cycle, the higher the rutting resistance of the asphalt mixture. The impact of the temperature on the rutting factors G*/sinδ of different binders is presented in Figure 5. The results show that increasing resins and asphaltenes increased the rutting factor, indicating an increase in rutting resistance. For example, the rutting factor of #7 asphalt binder at 64 °C was 1200% higher than of #11 asphalt binder. These results have also been noted in previous studies [49,50].

**Figure 6:**
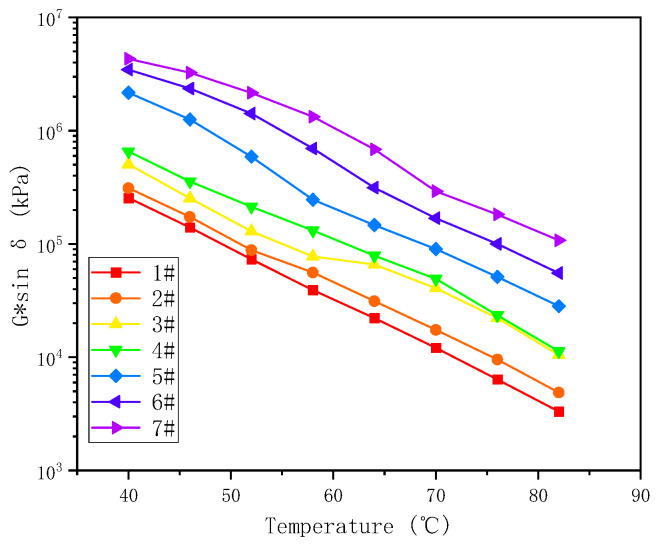
Variation of G*sinδ versus temperature.

A higher rutting factor can lead to the binder being susceptible to cracking risk under low- and medium-temperature conditions, and fatigue factor G*sinδ was introduced to characterize the ability of the asphalt binder to resist fatigue cracking due to the superpave performance grading (PG) method. The fatigue factor is the viscous component of the modulus (also known as the loss modulus), that is, the more energy the material losses during the loading process, the worse its corresponding anti-fatigue performance [51]. The variation curves of G*sinδ values with temperature for different binders are shown in Figure 6. As can be seen from Figure 6, the G*sinδ gradually decreased as the temperature increases, the viscosity and plasticity of the asphalt binder were greatly affected by temperature. With the increase of temperature, the asphalt binder changed from a “glass state” to a “liquid-like state” [35]. The viscous component increased, the elastic component decreased, and the fatigue resistance increased.

At the same temperature, the G*sinδ increased with increasing sample number, as follows: #7 > #6 > #5 > #4 > #3 > #2 > #1, indicating that the higher the number of resins and asphaltenes, the larger the G*sinδ. This in turn means that, with the increasing number of resins and asphaltenes, the glass transition temperature increases, which leads to an increase in stiffness at the same temperature [35]. The relevant literature points out that G*sinδ is the test result of a small strain and a small number of loading actions in the linear viscoelastic range and has nothing to do with the cumulative damage development under repeated loads. It cannot fully reflect the mechanical state and process of the actual pavement fatigue cracking and quantitative evaluation of the degree of asphalt fatigue [13].

### 3.2. Time Sweep (TS) Test Results

**Figure 7:**
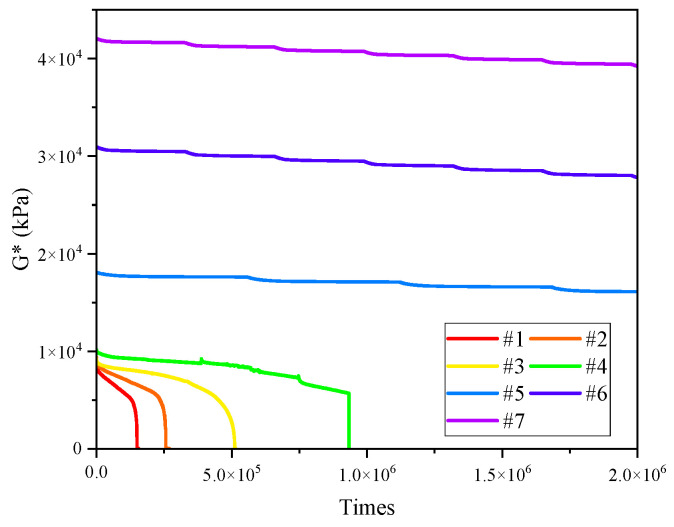
Complex modulus curve of TS test (0.1 Mpa).

**Figure 8:**
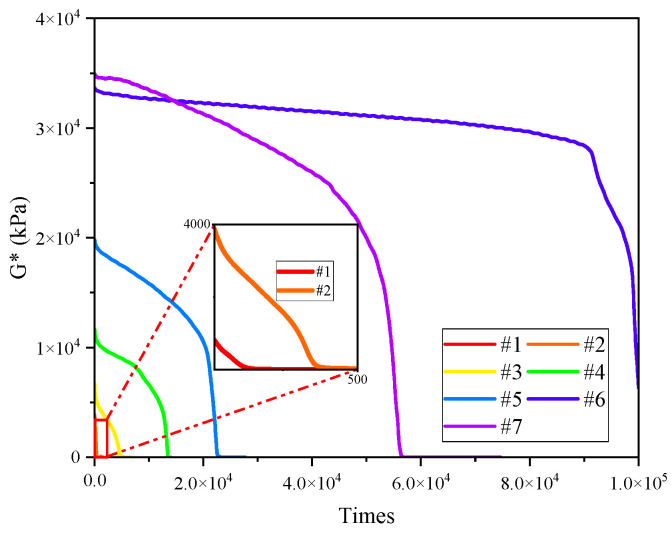
Complex modulus curve of TS test (0.2 Mpa).

The NCHRP 9-10 project proposes the application of TS test to evaluate the fatigue performance of asphalt [5]. This uses repeated application of loads on the sample, with a constant load amplitude and frequency, in order to interpret and represent the whole fatigue process. In the TS test, the fatigue life is determined by analyzing the decay law of macro technical indexes such as complex shear modulus and phase angle, which belongs to the asphalt fatigue life determination method based on the appearance method. The shear times (N_f50_) corresponding to the reduction of complex shear modulus to 50% of the initial value is used as the basis for fatigue failure, which corresponds to the determination of asphalt mixture fatigue (when the modulus is reduced to 50% of the initial value). The results of the TS tests of seven asphalt binders are shown in Figure 7 and Figure 8.

**Figure 9:**
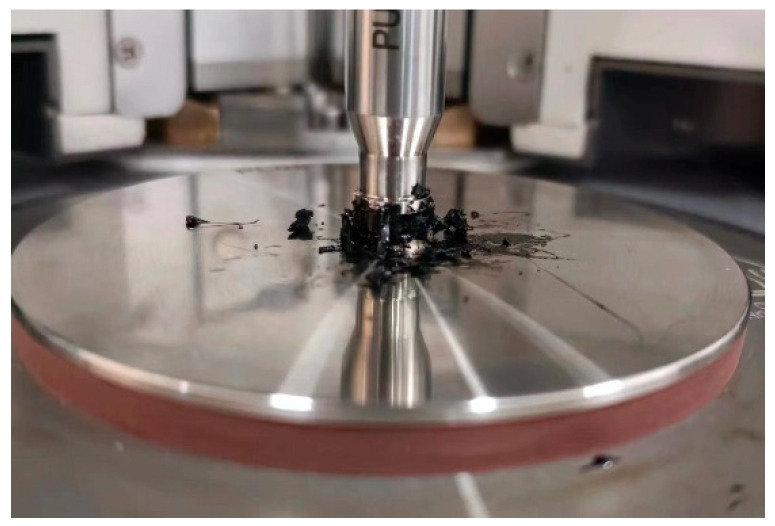
Photo of time sweep test of the #4 sample.

As can be seen from Figure 7, there were mutation points in the decay curve of the complex modulus of 4#, 5#, 6#, 7#, which was caused by the fact that the test time was too long, and that the DSR equipment could not complete the sweep test of the whole process in a single test due to the lack of computer memory. Thus, the test program needed to be repeated, which rendered the program discontinuous. Under a constant stress of 0.1 Mpa, with the increase of asphaltenes and resins, the initial complex modulus of binders increased, and the values of the #5, #6 and #7 samples were much higher than those of other binders, which is consistent with the temperature sweep results in Section 3.1. The complex modulus of all samples decreased with the increase of test time, while the decreasing trend was different. The complex modulus #5, #6 and #7 decreased slowly; when the number of stress cycles reached 2.0 × 10^6^, the decrease of complex modulus was still less than 50%, which means that these three binders had not showed obvious fatigue failure yet. This might be due to the fact that the samples contained enough asphaltenes and resins, as well as clusters composed of connected asphaltenes and resins, to form a high-strength spatial network structure, greatly increasing the strength of the asphalt colloidal structure. Under the stress of 0.1 Mpa, the internal damage of the colloidal structure became too small and inadequate to form a rapid accumulation phenomenon, and the complex modulus decreased slowly over a long test time. The complex modulus of the #4 sample, after 93,320 cycles, decreased from 10,145 kPa to 5702 kPa. A brittle fracture then occurred and the complex modulus quickly decreased to 0, as shown in Figure 9. For the #1, #2 and #3 samples, the complex modulus decreased slowly with the increasing test time. The Nf_50_ of #1 was 13,850, Nf_50_ of #2 was 23,988, and Nf_50_ of #3 was 46,374, which indicates that, under small stress, when the content of asphaltenes and resins increased, the strength of its colloidal structure increased, which could effectively increase the fatigue resistance of the binder.

Figure 8 shows the time sweep results under a constant stress of 0.2 Mpa. Compared with 0.1 Mpa, the results of 0.2 Mpa showed significant differences, the #5, #6 and #7 samples all showed fatigue failure, the complex modulus decreased gradually with the increasing test time, and after a certain time, the value quickly dropped to 0 before a brittle fracture occurred, all of which is similar to the situation of the #4 sample under 0.1 Mpa, seen in Figure 9. The Nf_50_ of the #1, #2, #3, and #4 samples were 60, 450, 4800, and 22,200, respectively. Compared with 0.1 Mpa, the fatigue life of samples decreased significantly, which indicates that, when the test stress increased, the micro-damage in the colloidal structure material increased, and the micro-damage accumulated rapidly with the increase of the test cycles. As a result, the fatigue failure of the binder was greatly advanced. Comparing the results of #5, #6 and #7, it can be seen that Nf_50_ first increased and then decreased with the increase of asphaltenes and resins and that the fatigue life did not increase with the increase of complex modulus. Under the stress of 0.2 Mpa, the cumulative damage of the #7 sample developed rapidly, and the complex modulus decreased rapidly, resulting in a lower Nf_50_ than #6.

In summary, when evaluating the fatigue performance using a time sweep test, different test stresses will lead to different conclusions. This is because different asphalt binders have different stress sensitivities, and the time sweep test can only reflect the accumulated fatigue damage inside the colloid structure under a single stress or strain. Therefore, in this test mode, the key to evaluating the fatigue performance is to select the appropriate test stress or strain for different asphalt binders. However, the pavement bears different loads in the actual service process, and the asphalt binders bear the stress or strain within a certain width domain, rather than the repeated action of the stress or strain of a single force value. Therefore, it is necessary to first study the cumulative damage of asphalt binders after experiencing the action of stress or strain from small to large and then to evaluate its anti-fatigue performance.

### 3.3. LAS Test Results

**Figure 10:**
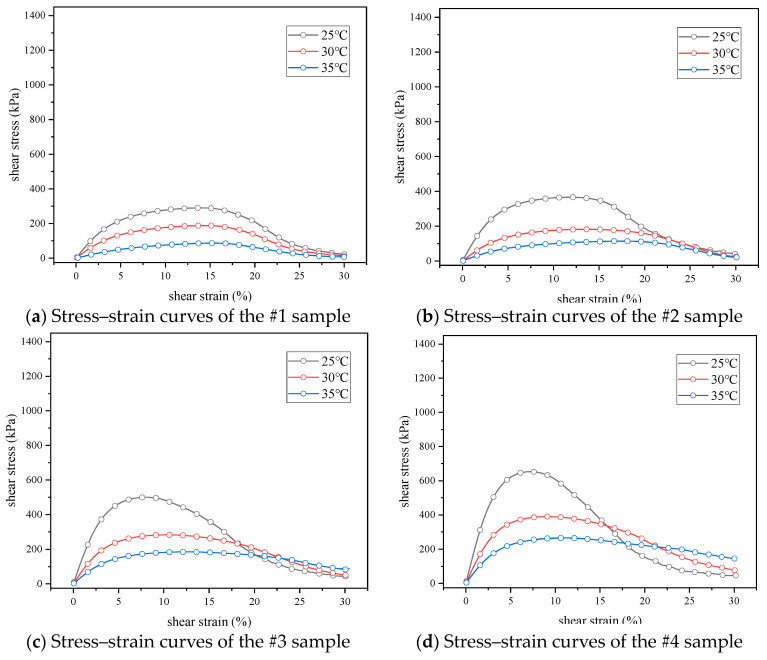

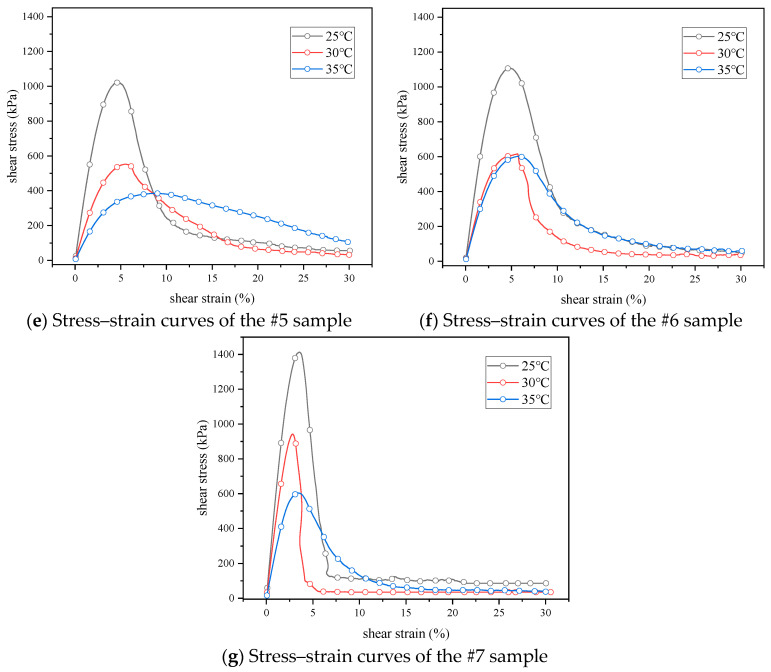
Stress–strain curves of seven kinds of asphalt binders.

In view of the deficiency of the rheometric and time sweep tests in evaluating the fatigue performance of an asphalt binder, the LAS test was used in this section to comprehensively evaluate the anti-fatigue performance of samples under repeated loads in a certain width domain. In order to explore the influence of temperature on the fatigue performance, LAS tests were carried out on seven samples under temperatures of 25 °C, 30 °C and 35 °C, the relevant stress–strain curves are shown in Figure 10. The peak stress can be understood as the yield threshold of the material under increased loading, where the phase angle drops after the material yielded [52,53]. As can be seen from Figure 10, there were stress peaks in all seven kinds of asphalt samples, with temperature having a significant effect on the stress–strain curve. With the increase of test temperature, the peak stress of each sample decreased gradually, and the corresponding shear strain increased gradually. For example, the peak stress of the #3 sample at 25 °C was 500 kPa and the peak strain was 7.8%, while at 35 °C, the peak stress was 185 kPa, and the peak strain was 12.5%. The peak stress decreased by 63% and the peak strain increased by 60%, which indicates that, with the increase of asphaltenes and resins, the yield threshold of the material increased with the increase of stiffness. As the sample number increased, the reduction of shear stress under different test temperatures changed from a smooth decrease to a sharp decrease at high shear strains. The stress–strain curves showed a steeper trend, as the smooth decrease indicates the plastic or viscous deformation of the specimen, while the sharp decrease indicates the fatigue failure of a brittle material. This phenomenon shows that rutting performance had been strengthened with the increase of asphaltenes and resins, and that, when the content of asphaltenes and resins was high, the fatigue properties of the sample may be reduced due to the sharp decrease at high shear strains. For the same sample, with the increase of the temperature, the state of the sample gradually changed. As an example, Figure 10g shows the transition from rutting (due to plasticity or viscosity) to fatigue failure (due to brittleness), 25 °C and 30 °C cause fatigue failure, while 35 °C causes rutting.

**Figure 11:**
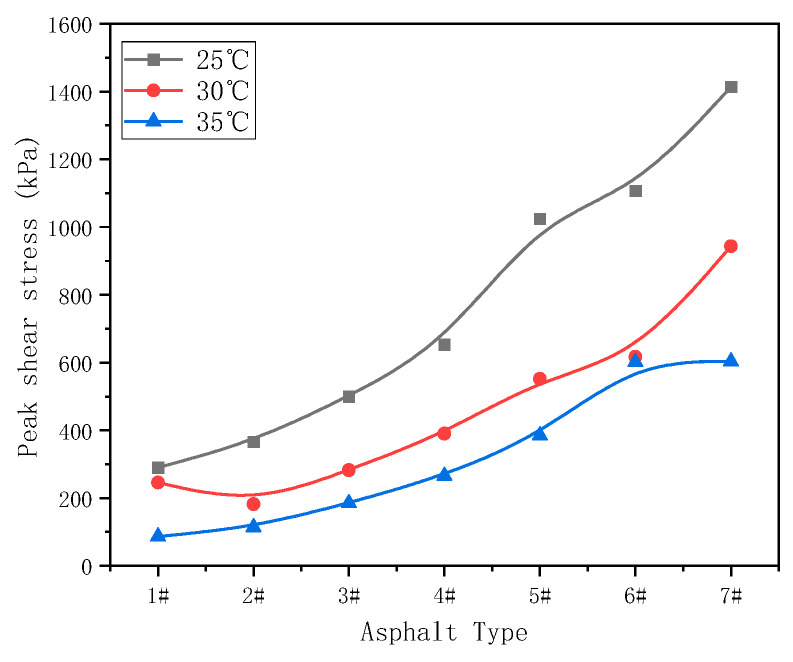
Peak stress at different temperatures.

**Figure 12:**
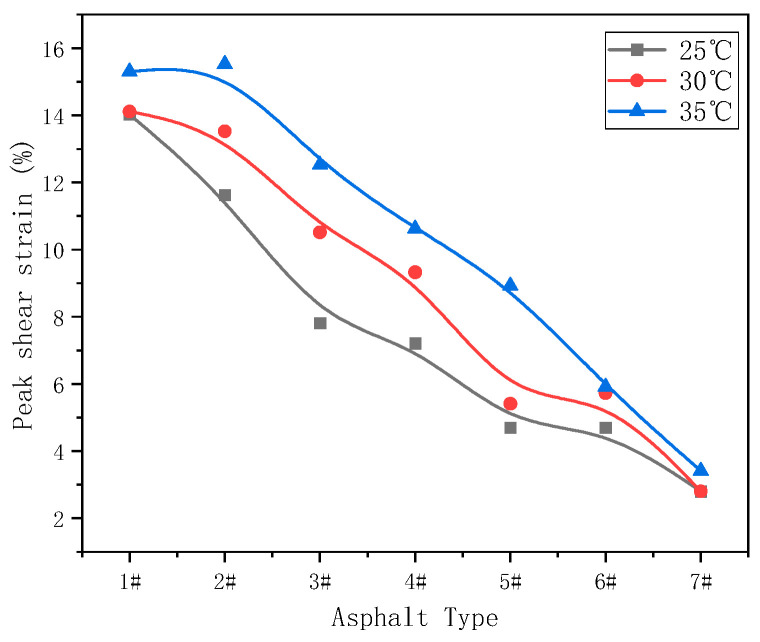
Peak strain at different temperatures.

By comparing the peak stress and peak strain of samples at different temperatures, shown in Figure 11 and Figure 12, respectively, it can be seen that the peak stress clearly increased, and that the peak strain decreased gradually at different test temperatures. This indicates that, with the increase of asphaltenes and resins, the binder became harder and its modulus increased, in turn indicating that a smaller shear strain would cause fatigue failure.

The peak range of the stress–strain curve represents the strain sensitivity of the asphalt binder. The wider the region, the lower the strain sensitivity. It can be seen from Figure 10 that, for the same asphalt, with the increase of test temperature, the peak range became wider and the strain sensitivity of asphalt decreased, which indicates that the modulus of the asphalt decreased gradually and could withstand a wider range of shear strain before fatigue failure occurred.

**Figure 13:**
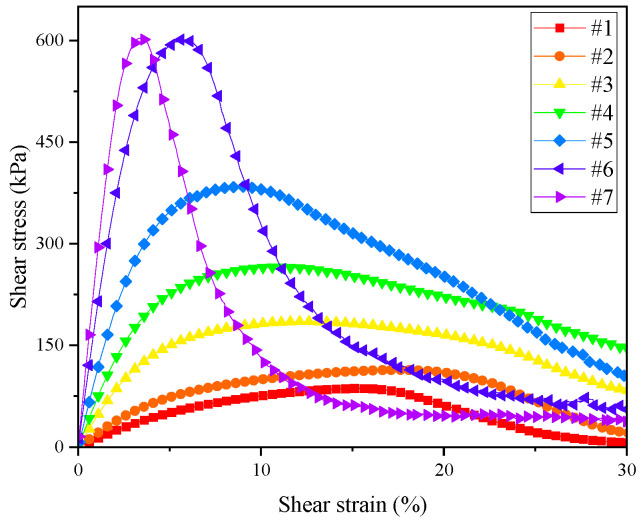
Stress–strain curves of seven samples at 35 °C.

Figure 13 showed the stress–strain curves of seven samples at 35 °C. With the increase of the sample number, the peak range of the stress–strain curves first increased and then decreased, indicating that the micelles composed by asphaltenes and the resins can form a spatial network structure, increasing the strength of the colloid structure and reducing the strain sensitivity. The peak ranges of #6 and #7 are extremely narrow, and the peak stress is much higher than that of other samples. Fatigue failure occurred within a short time after reaching the peak stress, which means that, with the increase of micelles, the peak range rapidly reduced, and the strain sensitivity increased significantly.

When LAS test was used to evaluate the fatigue performance of hard asphalt, the fatigue failure was accelerated by cyclic loading with a step-by-step increasing strain, which can fully reflect the state of hard asphalt under multiple loads. Thus, we were able to determine the suitable temperature and strain range of hard asphalt, which has important guiding significance for the rational utilization of hard asphalt.

**Figure 14:**
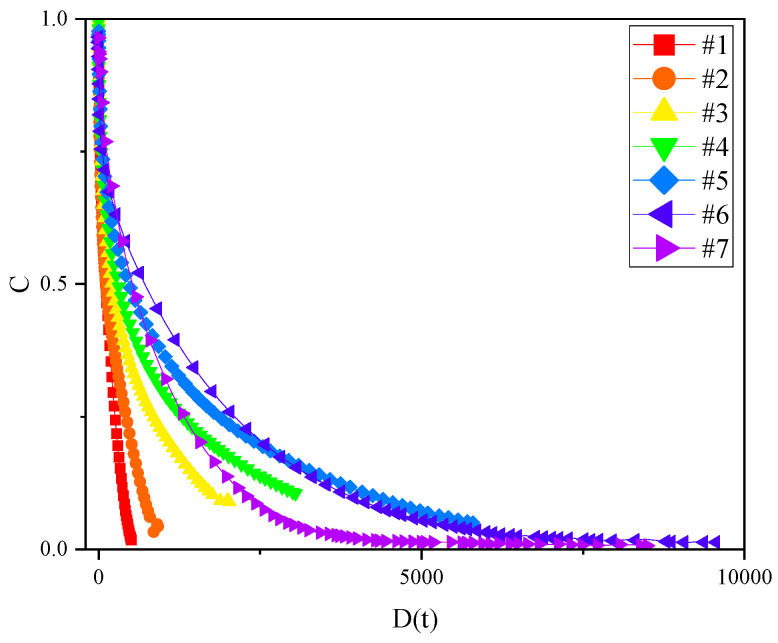
DC curves of the different samples at 35 °C.

The VECD model was used to draw the relationship between cumulative damage and the integrity of different samples at 35 °C, as shown in Figure 14. Abscissa D(t) represents the cumulative damage, and ordinate C (%) represents the integrity of samples. When C = 1, the asphalt sample was in an undamaged state; When C = 0, the asphalt sample had been completely damaged; when the cumulative damage parameter D was determined, the larger C was, the stronger was the ability of the material to resist damage. In the DC curve, in the area of low cumulative damage, the integrity parameters of the #1, #2, #3 and #4 samples decreased rapidly, while the integrity parameters of the #5, #6 and #7 samples decreased slowly, which means that the ability to resist fatigue damage of the #5, #6 and #7 samples was higher than that of #1, #2, #3 and #4 samples. This is contrary to the above conclusions, as can be seen from Figure 3. The modulus of the #5, #6 and #7 samples were significantly larger than that of other samples, and the calculated cumulative damage was much higher than that of other samples. As a result, in the same DC curve, the integrity parameter of the samples with small cumulative damage decreased rapidly, while the integrity parameter of the samples with large cumulative damage decreased slowly, distorting the physical index analysis of the DC curve. However, for the asphalt binders with the same level of complex modulus, such as the #6 and #7 samples, for which the cumulative damage D was the same, the integrity parameter of the #7 sample was smaller, indicating a lower integrity and worse fatigue resistance.

**Figure 15:**
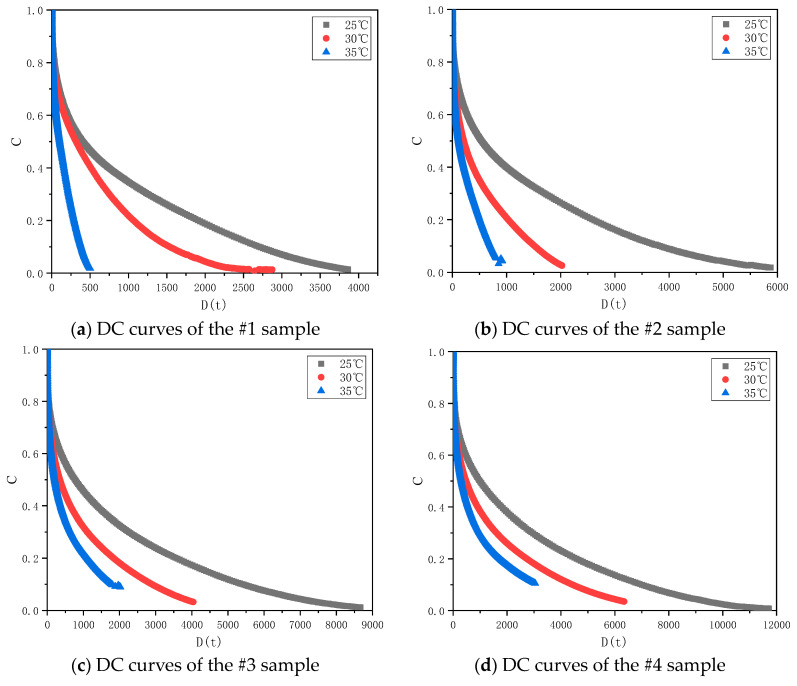

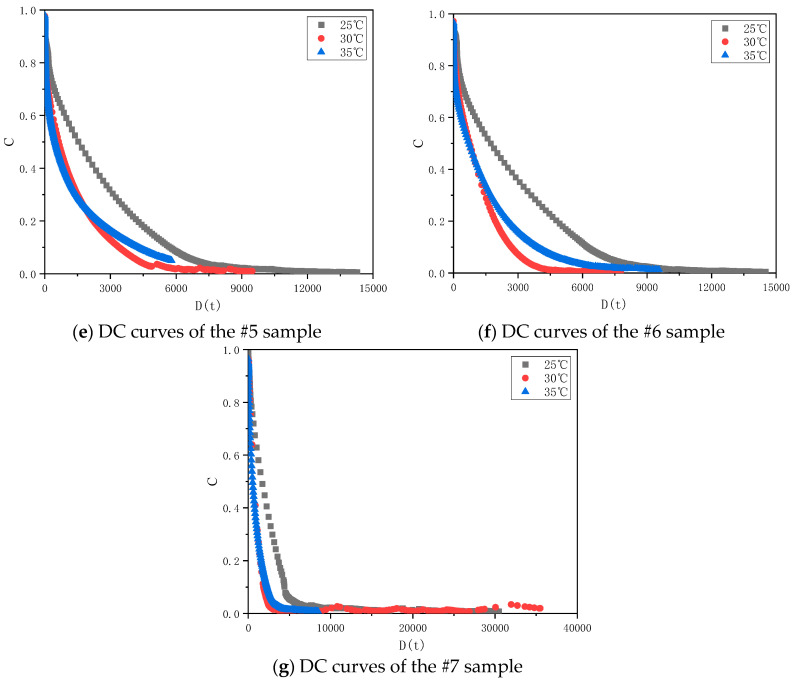
DC curves of different asphalt samples at 25 °C, 30 °C and 35 °C.

In order to explore the influence of different temperatures on the DC curve, the fatigue damage curves of seven samples at 25, 30 and 35 °C were drawn, as shown in Figure 15. With the increase of the test temperature, the DC curves showed different degrees of change. For the #1 and #2 samples, when the temperature increased from 25 °C to 35 °C, the C value decreased rapidly, as did the ability to resist fatigue damage. This was because the asphaltenes and resins of the #1 and #2 samples were inadequate to form a spatial network structure of sufficient strength. When the temperature increased, the stability of the colloidal structure decreased rapidly, and the internal structure of the asphalt was damaged rapidly under the action of the continuously increasing strain. For the #3 and #4 samples, with the increase of the test temperature, the C value decreased and the internal integrity of the binders decreased at the same cumulative damage D. Compared with the #1 and #2 asphalt binders, the decreasing trend of the C value at 35 °C slowed down, which indicates that, when the content of asphaltenes and resins increased to a certain amount, the clusters composed by asphaltenes and resins became interconnected to form a spatial network structure with a certain strength. When the test temperature increased, the spatial network structure helped the binders to resist the effect of the continuously increasing strain without rapid damage and fatigue failure.

For the #5, #6, and #7 samples, the DC curves at 30 °C and 35 °C were interwoven and the damage of the samples could not be well distinguished. This might be due to the way in which, when the number of clusters reaches a certain level, the spatial network structure they form them has enough strength to achieve a similar damage degree at both of the above temperatures.

**Figure 16:**
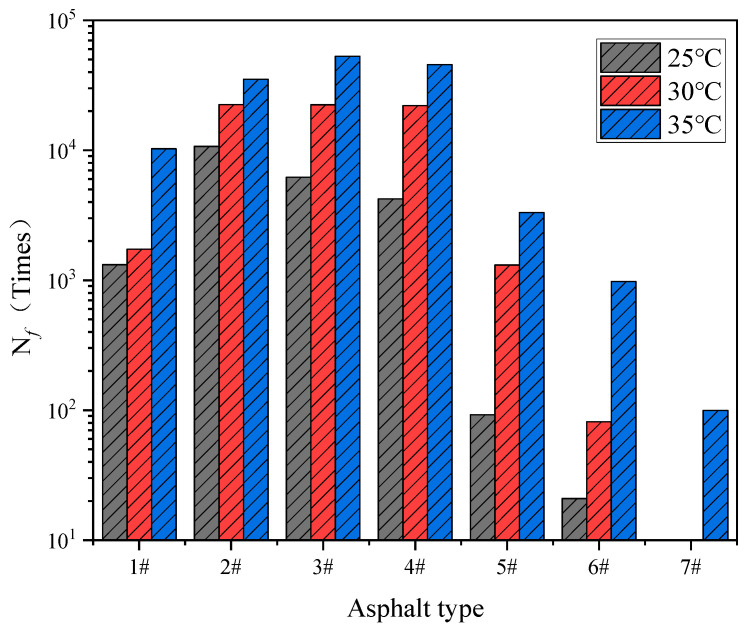
Fatigue life of the seven binders at 5% strain and different test temperatures.

In order to study the effect of temperature on fatigue life, Figure 16 show the fatigue life of each asphalt sample at three test temperatures under 5% strain. With the increase of the test temperature, the fatigue life increased to different degrees, and, due to the increase of the test temperature, the complex modulus decreased. Thus, the cumulative fatigue damage decreased, which would increase the calculated fatigue life.

As shown in Figure 10g, the stress–strain curve showed a rapid decline at 25 °C and 30 °C, meaning that the sample appeared to suffer a brittle fracture. Thus, the fatigue life of the #7 sample did not appear at 25 °C and 30 °C in Figure 16. By observing the change trend of the fatigue life of the seven samples, it can be seen that, with the increase of the sample number, the fatigue life generally showed a change law of first increasing and then decreasing, which might be related to the strain sensitivity of the colloidal structure and which is analyzed in the following section.

**Figure 17:**
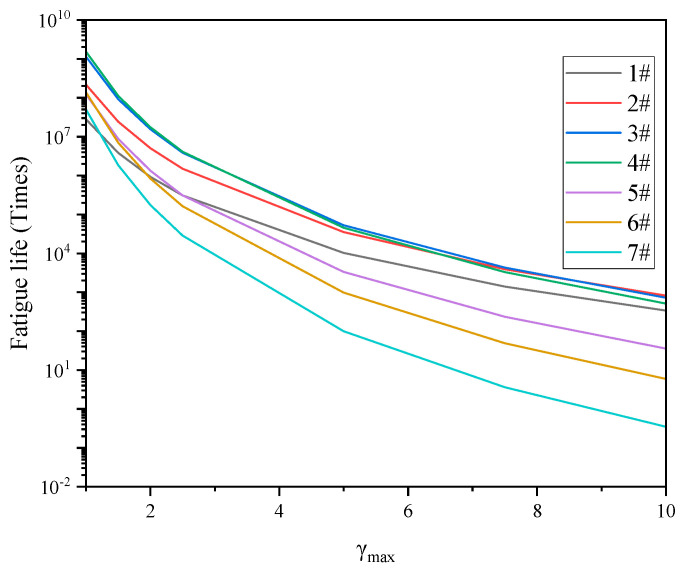
Fatigue life of different samples under different strains.

In order to analyze the effect of strain on the fatigue life, the trend of the fatigue life with different strains at 35 °C was plotted, as shown in Figure 17. It can be seen that, with the increase of shear strain, the cumulative damage increased, and the fatigue life showed a trend of gradually decreasing.

In the low strain zone, the fatigue life first increased and then decreased with the increase of sample number. With the increase of the strain, the fatigue life of different samples decreased in different trends. The fatigue life of the #5, #6, #7 samples decreased rapidly, and the decline rate was #7 > #6 > #5, which indicates that, if there were not enough aromatic and saturate fractions to provide deformation coordination, the spatial network structure composed by excessive asphaltenes and resins would quickly reduce the adaptability of the colloidal structure to the shear strains. The fatigue life of the #1 and #2 samples became gradually close to that of #3 and #4 samples. When the strain reached about 7%, the fatigue curves of the #2, #3 and #4 samples showed an interweaving phenomenon, which indicates that, when the strain was small, the colloid with good spatial network structure and deformation coordination ability could withstand the repeated action of small strain well and fewer fatigue damage would occur. With the increase of the strain, the deformation of the colloidal structure increased, the colloidal structure with more aromatics and saturates components obtained less fatigue damage than that of other samples due to its better deformation coordination, and their fatigue life became gradually close to the #3 and #4 samples. When the strain reached a certain level, the fatigue life of the #1 and #2 samples exceeded that of the #3 and #4 samples. The reason for this may be related to the strain sensitivity of the colloid structure. The above phenomena can be explained by the schematic diagram of the colloidal structure.

**Figure 18:**
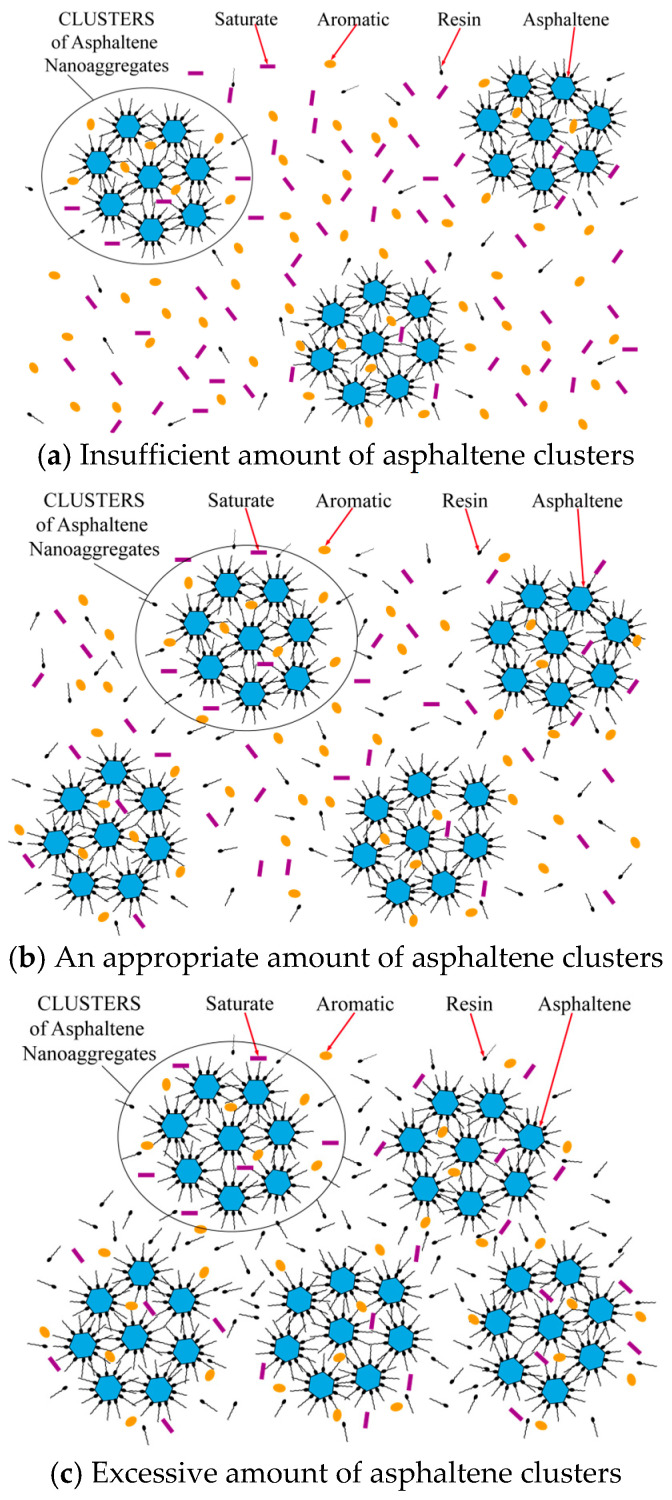
Diagram of colloid structure of asphalt with different SARA fractions.

Figure 18 shows the diagram of the colloidal structure of the clusters under different contact conditions. When the number of resins and asphaltenes was insufficient, as shown in Figure 18a, excessive aromatics and saturates dispersed to prevent clusters from connecting to each other and forming the spatial network structure, leading to the high stress sensitivity and lower fatigue life. When the number of resins and asphaltenes increased, the spatial network structure was gradually formed by the sufficient content of clusters, shown in Figure 18b. In this phase, the content of aromatics and saturates was moderate, which gave the colloidal structure a certain elastic recovery ability and deformation coordination performance at the same time. The strain sensitivity of asphalt binder was reduced, and the fatigue life under wider amplitude sweep was improved. In Figure 18c, as resin and asphaltene content continued to increase, the content of aromatics and saturates became too small, which led to the strain sensitivity of the colloidal structure increasing rapidly and the fatigue life decreasing greatly.

Through the analysis of the above test results, it can be found that 35% of the initial modulus, as the fatigue failure criterion, could well distinguish the fatigue properties of different hard asphalt binders at different temperatures, and the LAS test could be used to evaluate the fatigue characteristics of hard asphalt.

**Figure 19:**
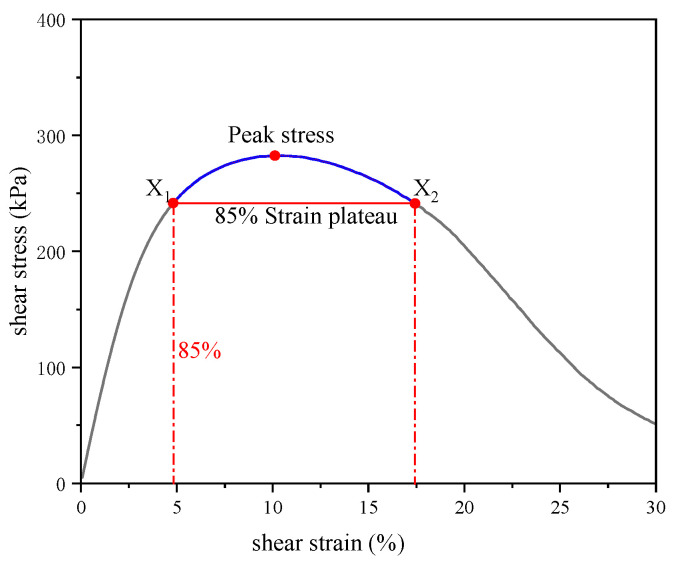
Schematic diagram of stress plateau in the stress–strain curve.

In order to better analyze the correlation between fatigue life and strain sensitivity, linear regression analysis of *N_f_* and strain sensitivity was carried out. In this study, the region of 85% of the peak stress in the stress–strain curve was defined as the strain plateau, as shown in Figure 19. The strain range of the strain plateau was used as the strain sensitivity parameter, and the fatigue life of the seven samples with *γ_max_* of 5% and 7.5% at 25 °C, 30 °C and 35 °C were linearly regressed with the strain sensitivity parameter.

**Figure 20:**
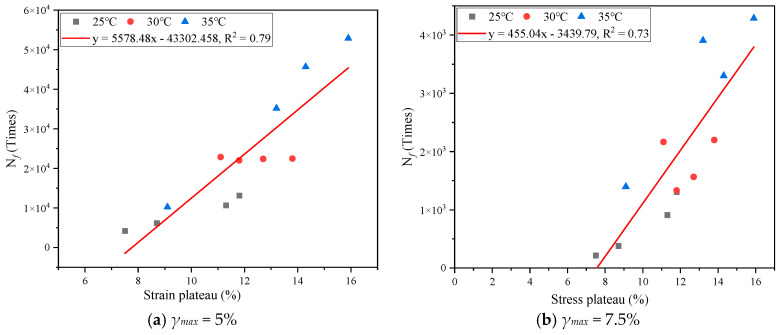
Linear regression analysis between fatigue life and strain sensitivity.

From the stress–strain curves, it can be seen that, with the increase of strain, the stress of the #5, #6 and #7 samples rapidly decreased to 0, the samples became completely fatigue damaged, and their fatigue life values at 25 °C and 30 °C became close to 0. In order to avoid the influence of this part of the data on linear regression, this part of the test data was eliminated in the regression analysis, and the linear regression situation was shown in Figure 20. R^2^ was 0.79 and 0.73, respectively, for 25 °C, 30 °C, and 35°C, which indicates that there was a good linear correlation between the fatigue life and the strain sensitivity of the colloidal structure at different calculated strains. In order to obtain hard asphalt with good fatigue properties, it is necessary to rationally configure the content of four components to reduce the strain sensitivity of its colloidal structure.

## 4. Conclusions

In this paper, SARA fractions were extracted from the same hard asphalt, and seven kinds of hard asphalt binders with different proportions of chemical components were formulated. The influence of different SARA fractions on the fatigue performance of the hard asphalt was discussed through rheological testing, time sweep testing and LAS testing. The key findings are summarized as follows:

(1) G*sinδ increases with the increasing number of resins and asphaltenes, which would reduce the anti-fatigue performances of the binder. This test reflects only a small strain and a limited number of loading cycles in the linear viscoelastic range, and larger strains and more test times must be adopted in order to better simulate the actual road conditions.
(2) For the same test samples, different test stresses will lead to different fatigue conclusions, and, when using the time sweep test, selecting the appropriate test stress or strain for different asphalt samples is key to evaluating the fatigue performance.
(3) The LAS test can fully reflect the actual stress state of asphalt binder under repeated loads, and effectively evaluate the fatigue performance of hard asphalt under different loads. When there is a significant difference in the order of magnitude of damage between different samples, the physical index analysis of DC curves may be distorted. Attention should be paid to the order of magnitude of cumulative damage when using DC curves to evaluate the fatigue damage.
(4) There is a good correlation between fatigue life and strain sensitivity as determined by linear regression analysis. To improve the fatigue characteristics of hard asphalt, it is necessary to adjust the proportion composition of SARA fractions reasonably to reduce the strain sensitivity of the colloidal structure.

Further research is necessary in order to optimize the current separation method to collect more SARA fractions and the influence mechanism of the four components on the fatigue properties of hard asphalt must be analyzed through a series of microscopic tests, such as atomic force microscopy (AFM), Fourier transform infrared (FTIR) spectroscopy, scanning electron microscope (SEM), etc.

## Figures and Tables

**Table 1:** Properties of virgin hard asphalt.

Test Items	Value	Testing Method	Contents (wt%)	Virgin Asphalts	Testing Method
Penetration 25 °C (0.1 mm)	29	T 0604	Saturates	17.11	T0618
Penetration index (PI)	0.92	T 0604			
Ductility (50 mm/min) 10 °C (cm)	1	T 0605	Aromatics	50.79	
Ductility (50 mm/min) 15 °C (cm)	8	T 0605			
Softening point (°C)	56	T 0606	Resins	16.88	
60 °C dynamic viscosity (Pa·S)	666.1	T 0620			
Solubility (%)	99.7	T 0607	Asphaltenes	15.22	

## Data Availability

Data are contained within the article.

## References

[1] Luo X., Luo R., Lytton R.L. (2013). Characterization of Asphalt Mixtures Using Controlled-Strain Repeated Direct Tension Test. J. Mater. Civ. Eng..

[2] Luo X., Luo R., Lytton R.L. (2015). Mechanistic modeling of healing in asphalt mixtures using internal stress. Int. J. Solids Struct..

[3] Ameri M., Nowbakht S., Molayem M., Mirabimoghaddam M.H. (2016). A study on fatigue modeling of hot mix asphalt mixtures based on the viscoelastic continuum damage properties of asphalt binder. Constr. Build. Mater..

[4] Wang C., Zhang H., Castorena C., Zhang J., Kim Y.R. (2016). Identifying fatigue failure in asphalt binder time sweep tests. Constr. Build. Mater..

[5] Bahia H.U., Hanson D.I., Zeng M., Zhai H., Khatri M.A., Anderson R.M. (2001). Characterization of Modified Asphalt Binders in Superpave Mix Design.

[6] Yan C., Yuan L., Yu X., Ji S., Zhou Z. (2022). Characterizing the fatigue resistance of multiple modified asphalts using time sweep test, LAS test and elastic recovery test. Constr. Build. Mater..

[7] (1999). Asphalt—Road Base Courses: Road Base High Modulus Asphalt Concrete—Definition—Classification—Characteristics—Fabrication—Laying.

[8] Botes B.H. (2016). Characterisation of High Modulus Asphalt (EME) Mixes, focussing on Flexural Response and Fatigue. Ph.D. Thesis.

[9] Li G., Chen Z., Tan Y., Cong X., Dong Y., Xiao S. (2023). Experimental and molecular dynamics simulation of hard asphalt microstructure. Constr. Build. Mater..

[10] Dias M., Petho L., Denneman E., Beecroft A. (2017). High Modulus High Fatigue Resistance Asphalt (EME2) Technology Transfer: Final Report. https://trid.trb.org/view/1480565.

[11] Petho L., Bryant P. High modulus asphalt (EME2) pavement design in Queensland. Proceedings of the 2015 AAPA International Flexible Pavements Conference.

[12] Zhou F.J., Mogawer W., Li H.S., Andriescu A., Copeland A. (2013). Evaluation of Fatigue Tests for Characterizing Asphalt Binders. J. Mater. Civ. Eng..

[13] Bahia H.U., Zhai H., Zeng M., Hu Y., Turner P. (2001). Development of binder specification parameters based on characterization of damage behavior. J. Assoc. Asphalt Paving Technol..

[14] Bahia H.U., Zhai H., Bonnetti K., Kose S. (1999). Non-linear viscoelastic and fatigue properties of asphalt binders. J. Assoc. Asphalt Paving Technol..

[15] Masad E., Castelo Branco V.T.F., Little D.N., Lytton R. (2008). A unified method for the analysis of controlled-strain and controlled-stress fatigue testing. Int. J. Pavement Eng..

[16] Daniel J.S., Kim Y.R. (2002). Development of a simplified fatigue test and analysis procedure using a viscoelastic, continuum damage model. J. Assoc. Asph. Paving Technol..

[17] Bhasin A., Castelo Branco V.T., Masad E., Little Dallas N. (2009). Quantitative Comparison of Energy Methods to Characterize Fatigue in Asphalt Materials. J. Mater. Civ. Eng..

[18] Kim Y.R., Lee H.J., Little D.N. (1997). Fatigue characterization of asphalt concrete using viscoelasticity and continuum damage theory. J. Assoc. Asph. Paving Technol..

[19] Hicks R.G., Finn F.N., Monismith C.L., Leahy R.B. (1993). Validation of SHRP binder specification through mix testing. J. Assoc. Asph. Paving Technol..

[20] Yan J., Leng Z., Ling C., Zhu J., Zhou L. (2020). Characterization and comparison of high-modulus asphalt mixtures produced with different methods. Constr. Build. Mater..

[21] Lei Z., Bahia H., Yi-qiu T., Ling C. (2017). Effects of refined waste and bio-based oil modifiers on rheological properties of asphalt binders. Constr. Build. Mater..

[22] Ling C., Arshadi A., Bahia H. (2017). Importance of binder modification type and aggregate structure on rutting resistance of asphalt mixtures using image-based multi-scale modelling. Road Mater. Pavement Des..

[23] Ogbo C., Kaseer F., Oshone M., Sias J.E., Martin A.E. (2019). Mixture-based rheological evaluation tool for cracking in asphalt pavements. Road Mater. Pavement Des..

[24] Zhou L., Huang W., Zhang Y., Lv Q., Yan C., Jiao Y. (2020). Evaluation of the adhesion and healing properties of modified asphalt binders. Constr. Build. Mater..

[25] Hintz C., Velasquez R., Johnson C., Bahia H. (2011). Modification and Validation of Linear Amplitude Sweep Test for Binder Fatigue Specification. Transp. Res. Rec. J. Transp. Res. Board.

[26] Estimating Damage Tolerance of Asphalt Binders Using the Linear Amplitude Sweep. AASHTO: Washington, DC, USA, 2014. https://uwmarc.wisc.edu/files/linearamplitudesweep/AASHTO-TP101-LAS-May-2013-v2.pdf.

[27] Mansourian A., Goahri A.R., Khosrowshahi F.K. (2019). Performance evaluation of asphalt binder modified with EVA/HDPE/nanoclay based on linear and non-linear viscoelastic behaviors. Constr. Build. Mater..

[28] Saboo N. (2020). New Damage Parameter for Fatigue Analysis of Asphalt Binders in Linear Amplitude Sweep Test. J. Mater. Civ. Eng..

[29] Zou G., Zhuo R., Sun X., Luo J. (2020). Effects of crude oil on the performances of hard asphalt and its mixtures. Int. J. Pavement Eng..

[30] Quan X., Chen C., Ma T., Zhang Y. (2024). Performance evaluation of rapeseed oil-based derivatives modified hard asphalt binders: Towards greener and more sustainable asphalt additives. Constr. Build. Mater..

[31] Qin R.J., Li Y.Z. (2009). Study the test methods and pavement performance indexes about hard asphalt binder material. Proceedings of the 2009 International Conference on Measuring Technology and Mechatronics Automation.

[32] Chen Y., Wang H.N., Xu S.B., You Z.P. (2020). High modulus asphalt concrete: A state-of-the-art review. Constr. Build. Mater..

[33] Khiavi A.K., Naseri S. (2019). The effect of bitumen types on the performance of high-modulus asphalt mixtures. Pet. Sci. Technol..

[34] Zhang Y.N., Cheng H.L., Sun L.J., Liu L.P., Hu Y. (2021). Determination of volumetric criteria for designing hard asphalt mixture. Constr. Build. Mater..

[35] Yadykova A.Y., Strelets L.A., Ilyin S.O. (2023). Infrared Spectral Classification of Natural Bitumens for Their Rheological and Thermophysical Characterization. Molecules.

[36] Yadykova A.Y., Ilyin S.O. (2022). Compatibility and rheology of bio-oil blends with light and heavy crude oils. Fuel.

[37] Mirwald J., Werkovits S., Camargo I., Maschauer D., Hofko B., Grothe H. (2020). Investigating bitumen long-term-ageing in the laboratory by spectroscopic analysis of the SARA fractions. Constr. Build. Mater..

[38] Mikhailenko P., Baaj H. (2019). Comparison of Chemical and Microstructural Properties of Virgin and Reclaimed Asphalt Pavement Binders and Their Saturate, Aromatic, Resin, and Asphaltene Fractions. Energy Fuels.

[39] Koots J., Speight J.G. (1975). Relation of petroleum resins to asphaltenes. Fuel.

[40] (2011). Standard Test Methods of Bitumen and Bituminous Mixtures for Highway Engineering.

[41] (2023). Standard Specification for Performance-Graded Asphalt Binder.

[42] Hamid A., Baaj H., El-Hakim M. (2022). Rutting Behaviour of Geopolymer and Styrene Butadiene Styrene-Modified Asphalt Binder. Polymers.

[43] Li H., Luo X., Zhang Y.Q., Xu R.Q. (2021). Stochastic fatigue damage in viscoelastic materials using probabilistic pseudo J-integral Paris’ law. Eng. Fract. Mech..

[44] Elkashef M., Williams R.C. (2017). Improving fatigue and low temperature performance of 100% RAP mixtures using a soybean-derived rejuvenator. Constr. Build. Mater..

[45] Gu H., Zhu Y., Zhu Y., Chu L., Peng Q., Chen Q. (2023). Rheological Properties and Microscopic Characterization of Delayed Decay-Modified Asphalt Based on UV Ageing. Adv. Mater. Sci. Eng..

[46] Baditha A.K., Muppireddy A.R., Kusam S.R. (2022). A Study on Chemical Composition, Colloidal Stability, and Rheological Properties of Ethylene Vinyl Acetate–Modified Binders. J. Mater. Civ. Eng..

[47] Sulaimon A.A., A/L Rajan H., Qasim A., Christiana N.P., Murungi P.I. (2023). Developing new correlations for asphaltene deposition involving SARA fractions and colloidal instability index. J. Pet. Sci. Eng..

[48] Stankiewicz A.B., Flannery M.D., Fuex N.A. Prediction of asphaltene deposition risk in E&P operations. Proceedings of the 3rd International Symposium on Mechanisms and Mitigation of Fouling in Petroleum and Natural Gas Production.

[49] Wang T., Wang J., Hou X., Xiao F. (2019). Effects of SARA fractions on low temperature properties of asphalt binders. Road Mater. Pavement Des..

[50] Xu Y., Zhang E., Shan L. (2019). Effect of SARA on Rheological Properties of Asphalt Binders. J. Mater. Civ. Eng..

[51] Anderson D.A., Kennedy T.W. (1993). Development of Shrp Binder Specification (with Discussion). J. Assoc. Asph. Paving Technol..

[52] Wang C., Castorena C., Zhang J., Richard Kim Y. (2015). Unified failure criterion for asphalt binder under cyclic fatigue loading. Road Mater. Pavement Des..

[53] Cao W., Wang C. (2018). A new comprehensive analysis framework for fatigue characterization of asphalt binder using the Linear Amplitude Sweep test. Constr. Build. Mater..

